# The impact of swine diseases on total factor productivity of pig farms of different scales in China

**DOI:** 10.1186/s40813-025-00484-z

**Published:** 2026-01-24

**Authors:** Zhen Xu, Xiangdong Hu, Fengqi Xu, Haizhao Zhang, Hui Zhou

**Affiliations:** Institute of Agricultural Economics and Development, Chinese Academy of Agricultural Sciences, Beijing, China

**Keywords:** Animal health economics, DEA-malmquist index, Two-way fixed effects model, Scale heterogeneity, China swine industry

## Abstract

**Background:**

Animal diseases threaten pig production by reducing growth efficiency and farm profitability. Total factor productivity (TFP), reflecting technological progress, organizational improvement, and resource allocation, provides a comprehensive measure of farm performance. Yet, the impact of diseases on TFP in pig farming remains unclear. This study examines the effects of animal diseases on TFP across different farm sizes in China using provincial panel data from 2007 to 2020.

**Results:**

A 1% increase in disease cases raises TFP by 0.018 units in large-scale farms, reduces it by 0.041 units in small-scale farms, and shows no significant effect for medium-scale farms. Mechanism analysis indicates that diseases mainly affect TFP through technical change, while heterogeneity analysis shows stronger effects in major pig-producing regions.

**Conclusions:**

Diseases, though adverse shocks, have spurred technological progress and TFP in large-scale farms but significantly constrained TFP in small-scale farms. These findings underscore the need to promote technological innovation, account for scale heterogeneity in policy design, and strengthen the resilience of small- and medium-scale farms to ensure sustainable development of the swine industry.

**Supplementary Information:**

The online version contains supplementary material available at 10.1186/s40813-025-00484-z.

## Background

Animal diseases can directly lead to a decline in animal productivity, resulting in production losses or reduced production efficiency [1]. For swine, major infectious diseases such as African swine fever and porcine reproductive and respiratory syndrome (PRRS) are highly likely to result in substantial economic losses. After the outbreak of African swine fever in China, the number of pigs in stock decreased by 27.5% year-on-year by the end of 2019, and pork production decreased by 21.3%.^1^ The reduction in supply led to a sharp increase in prices, with pork prices reaching a peak of 59.88 yuan/kg in November 2019, an increase of 174.5% compared to the average in 2018^2^. Although the African swine fever epidemic has eased in recent years, swine diseases represented by African swine fever and PRRS remain major threats to pig farms. Studies have shown that outbreaks of PRRS lead to a decline in the reproductive health of sows, significantly reducing the production of weaned piglets, affecting the growth rate and efficiency of piglets, and making it difficult to recover to healthy levels. This production loss results in a decline in pig production efficiency, with an average reduction of 1.92 piglets per sow [2, 3]. In addition to its impact on sows and piglets, PRRS also affects the growth of fattening pigs, leading to a significant decline in growth performance, feed intake, and feed efficiency [4]. Additionally, porcine epidemic diarrhea can also cause a decline in growth performance, feed efficiency, and digestibility in pigs [5, 6], leading to a decrease in pig production efficiency and economic losses for producers [7].

When evaluating the performance of pig farms, production efficiency measured by traditional input factors such as feed and labor is certainly important. However, from a long-term development perspective, production competitiveness, risk resilience, and growth potential are the key determinants of the development quality and sustainability of pig farms. As a core economic indicator, Total Factor Productivity (TFP) captures the overall efficiency gains derived from technological progress, managerial improvements, scale effects, and better resource allocation, after controlling for conventional input factors.

According to the Cobb–Douglas production function, output can be expressed as a functional form of traditional input factors, namely:

1 $$Y=A\cdot{K}^{\alpha\:}\cdot{L}^{\beta\:}\cdot{X}^{\gamma\:}$$

Here, $\:Y\:$ denotes output; $\:K$, $\:L$, and $\:X$ represent capital, labor, and other input factors (such as land or feed), respectively; $\:\alpha\:$, $\:\beta\:$, and $\:\gamma\:$ are the output elasticities of these inputs, reflecting their relative contributions to output; and A represents TFP. Taking the logarithmic derivative of the production function yields the standard growth decomposition equation:

2 $$\varDelta{Y}=\alpha\:\cdot\varDelta{K}+\beta\:\cdot\varDelta{L}+\gamma\:\cdot\varDelta{X}+\varDelta{A}$$

Among these components, the first few terms capture the contribution of traditional input factors to output, whereas ∆A represents the portion of output growth that cannot be explained by input quantities. From the perspective of growth accounting, Eq. (2) can be further expressed in a simplified form as:

3 $$\varDelta{Y}=\varDelta{Efficiency}+\varDelta{A}$$

Among these components, ∆Efficiency captures the changes in production efficiency generated by traditional factor inputs, whereas ∆A reflects the changes in TFP driven by technological progress and other non-traditional inputs. In practice, pig farms may enhance TFP—and thereby improve overall production efficiency—by adopting advanced technologies such as digital farming solutions, precision nutrition programs, and biological breeding technologies, combined with highly efficient enterprise-level and intensive management models.

As noted above, existing research in the natural sciences has demonstrated that pig diseases lead to reductions in production efficiency. However, scholars in the field of economics have paid comparatively little attention to the relationship between swine diseases and TFP of pig farms.

Currently, research on TFP in animal husbandry mainly focuses on two aspects: one is the measurement and decomposition of TFP for different animal products, and the other is the analysis of the factors influencing TFP. Existing studies have shown that technological progress is widely regarded as the core driving force behind TFP growth. In European countries, TFP in dairy cattle and meat sheep farms has continuously improved, primarily relying on technological innovation [8–11]. In China, the early growth of TFP in pig production relied mainly on the expansion of input factors, while in recent years it has gradually shifted towards relying on technological progress and efficiency improvement [12–14]. There is a significant difference in TFP between farms of different sizes, with free-range and small-scale farms showing a downward trend, while medium- and large-scale farms have shown steady growth [15–17]. In terms of influencing factors, the development of industry consulting services, reforms in government regulation, the emergence of income insurance, and contracted production models, as well as technological advancements such as the use of precision feeding strategies, have promoted the increase in TFP of animal products [18–22]. In the Chinese context, macro factors such as regional economic development level and the level of animal husbandry development can have spillover effects on local pig TFP [23]. Micro factors such as breeding subsidies, specialized division of labor, education level, technical training, farming scale, farming density, farming risks, and mechanization level all have a significant impact on pig TFP [24–27].

It is worth noting that research on the impact of animal diseases on TFP is still relatively limited. Limited evidence suggests that African swine fever has led to a decrease in technical efficiency in large-scale farms [28], and the occurrence of zoonotic diseases is also significantly related to TFP losses [29]. This suggests that diseases, as external shocks, may have complex effects on TFP, but the heterogeneity of scale and the mechanisms of impact still need further exploration, providing a theoretical basis for this study. Therefore, this paper aims to empirically analyze and quantify the impact of swine diseases on TFP in pig farms of different sizes in China, and analyze the underlying mechanisms of this impact. The main contributions of this study are as follows. First, from the perspective of TFP, it systematically assesses the impact of disease shocks on the TFP of the pig farming industry, thereby enriching the quantitative literature on the economic consequences of animal diseases. Second, by incorporating the perspective of scale heterogeneity, the study further uncovers differences in TFP responses to disease shocks among pig farms of varying sizes, providing more nuanced evidence for understanding the relationship between animal diseases and farm-level TFP.

## Materials and methods

### Sample selection

**Fig. 1:**
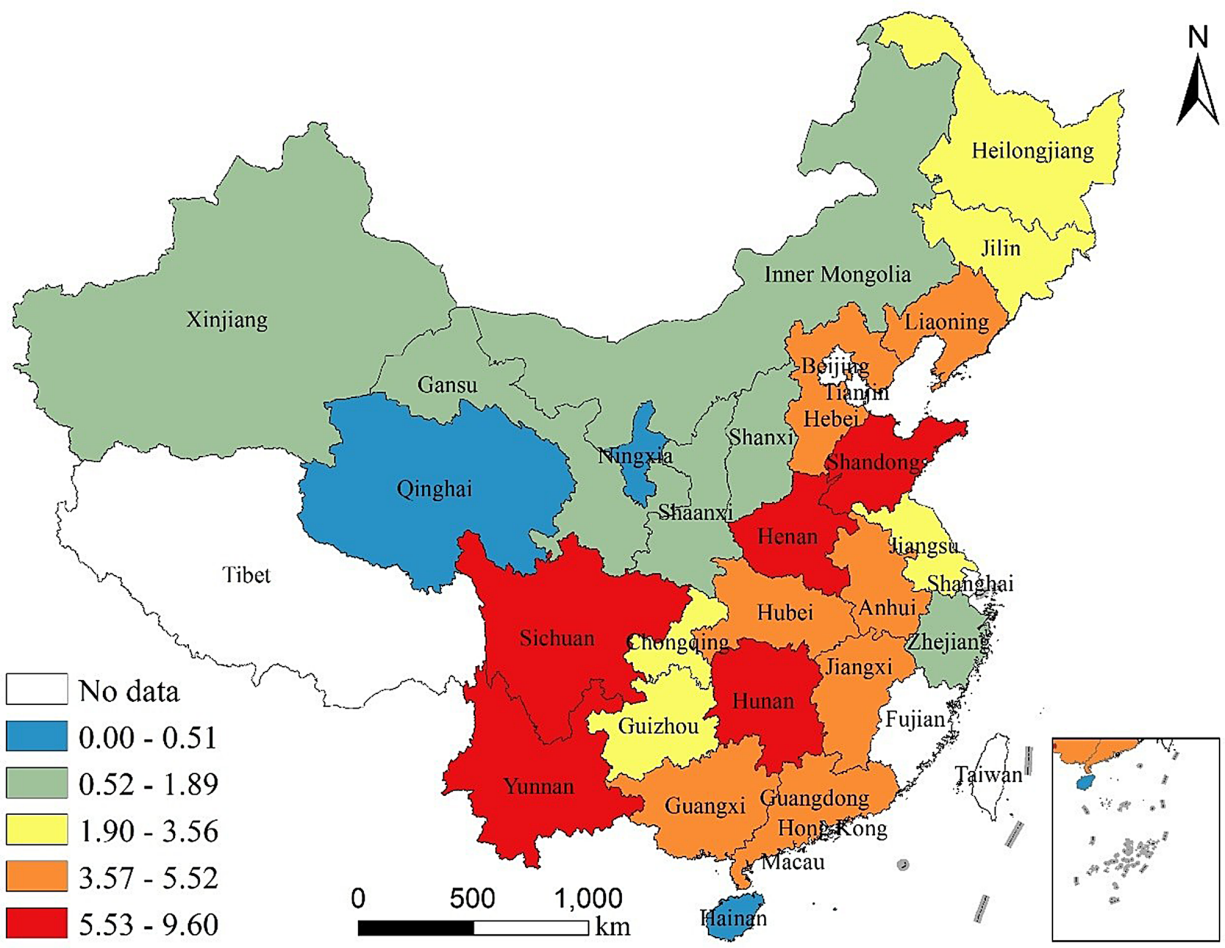
Sample provinces and their share of national pork production. Map Review Number: GS(2024)0650

This study selects 26 provinces (autonomous regions and municipalities) in China as the research sample, constructing a provincial panel dataset for China from 2007 to 2020, as shown in Fig. 1,^3^. The selected provinces account for more than 96% of the national pork production, including 18 major pig production provinces and 8 non-major production provinces. To ensure the accuracy of the estimation results, this study excludes provinces with low pork production and severe data missing, such as Beijing, Tianjin, and Tibet.

The standards for classifying pig farms of different sizes and the data required for calculating TFP come from the “National Agricultural Products Cost and Return Compilation” (hereinafter referred to as the “Compilation”) published by the National Development and Reform Commission of China. This data is compiled from survey data collected from 31 provinces, 1,553 counties, and over 66,000 farm households, covering production and cost-benefit data for more than 50 agricultural products, comprehensively reflecting the production and business conditions of agricultural products.

In the “Compilation,” pig farms are classified into four categories based on the designed maximum stocking capacity ($\:Q$) for statistical purposes: free-range households ($\:Q\le\:30$), small-scale ($\:30<Q\le\:100$), medium-scale ($\:100<Q\le\:1000$), and large-scale ($\:Q>1000$). This classification standard is uniformly formulated by the National Development and Reform Commission, with authority and continuity, providing a basis for objectively comparing the efficiency of farms of different sizes. It should be noted that due to the limited coverage of free-range households and the high number of missing key indicators, making it difficult to ensure the validity of the estimates, this study does not consider free-range households and focuses only on scaled farms.

### Methods

The research methods involved in this study are mainly divided into two parts: the DEA-Malmquist index method for measuring TFP in pig farms, and the Two-way fixed effects model for analyzing the relationship between diseases and TFP in pig farms.

#### DEA-malmquist index

**Fig. 2:**
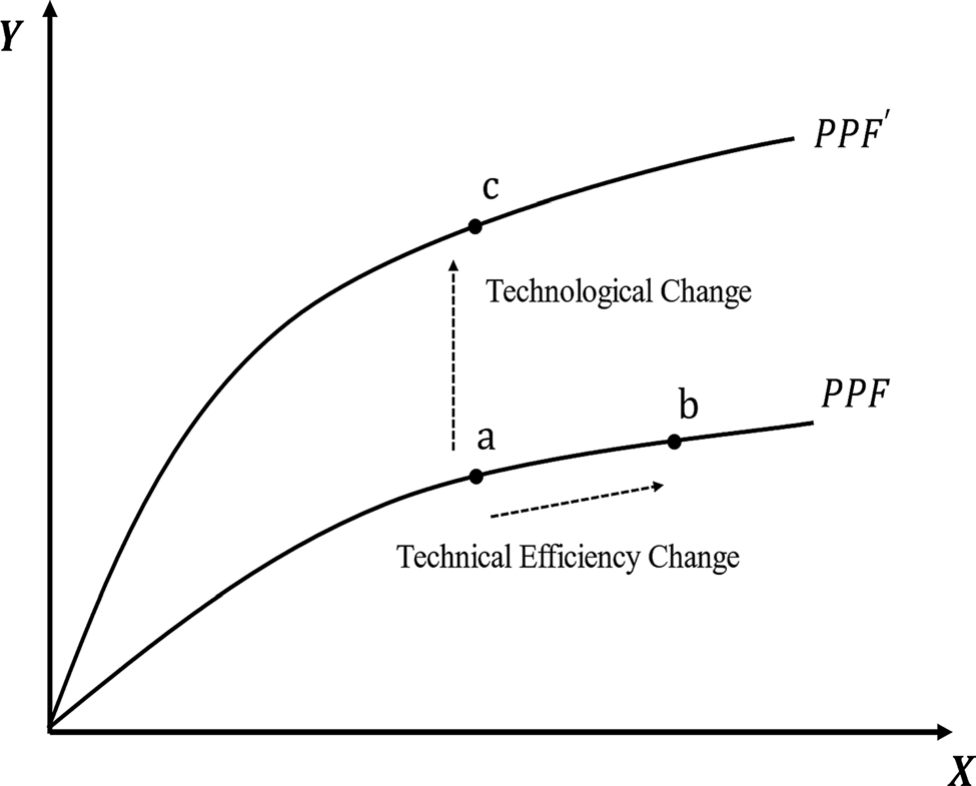
Decomposition of total factor productivity into technical efficiency change and technological change. Note: This figure illustrates the sources of total factor productivity change based on the production possibility frontier (PPF). The horizontal axis (X) represents input factors, and the vertical axis (Y) denotes output. Under a given technology, the movement from point *a* to point *b* reflects technical efficiency change, while the outward shift of the frontier from $\:PPF$ to $\:{PPF}^{{\prime\:}}$ and the movement from point *a* to point *c* indicate technological change

As introduced earlier, the concept of TFP and its distinction from production efficiency have been discussed. More specifically, TFP can be decomposed into two components: technical efficiency (TE) and technological change (TC). Improvements in TE are reflected in shifts of the actual production point relative to the production possibility frontier, typically associated with enhancements in resource allocation and management practices—for instance, the movement from point a to point b in Fig. 2. TC, on the other hand, refers to an outward shift of the production possibility frontier, generally linked to the adoption of new technologies or innovations, as illustrated by the movement from point a to point c in Fig. 2.

This study is based on the DEA-based Malmquist index, using the ratio of distance functions to calculate input-output efficiency. Referencing the study by Li et al. [30], this paper introduces two classic formulas to explain the principles of the Malmquist productivity index.

4 $$\begin{aligned}&{M}_{i,t+1}\left({x}_{i}^{t},{y}_{i}^{t},{x}_{i}^{t+1},{y}_{i}^{t+1}\right)\cr&\quad=\sqrt{\frac{{D}_{i}^{t}\left({x}_{i}^{t+1},{y}_{i}^{t+1}\right)}{{D}_{i}^{t}\left({x}_{i}^{t},{y}_{i}^{t}\right)}\cdot\:\frac{{D}_{i}^{t+1}\left({x}_{i}^{t+1},{y}_{i}^{t+1}\right)}{{D}_{i}^{t+1}\left({x}_{i}^{t},{y}_{i}^{t}\right)}}\end{aligned}$$

In Eq. (4), $\:{x}_{i}^{t}$ and $\:{x}_{i}^{t+1}$ represent the input vectors for region $\:i$ in periods $\:t$ and $\:t+1$, respectively; $\:{y}_{i}^{t}$ and $\:{y}_{i}^{t+1}$ represent the output vectors for region $\:i$ in periods $\:t$ and $\:t+1$, respectively; $\:{D}_{i}^{t}({x}_{i}^{t},{y}_{i}^{t})$ and $\:{D}_{i}^{t}({x}_{i}^{t+1},{y}_{i}^{t+1})$ represent the distance functions for the production points in periods $\:t$ and $\:t+1$, with the technology $\:{\:T\:}^{t}$ from period $\:t$ as the reference.

5 $$\:\begin{aligned}&{M}_{i,t+1}\left({x}_{i}^{t},{y}_{i}^{t},{x}_{i}^{t+1},{y}_{i}^{t+1}\right)\cr&\quad=\underbrace {\frac{{D}_{i}^{t+1}\left({x}_{i}^{t+1},{y}_{i}^{t+1}\right)}{{D}_{i}^{t}\left({x}_{i}^{t},{y}_{i}^{t}\right)}}_{{TE}_{i}^{t+1}}{{\sqrt{\underbrace {\frac{{D}_{i}^{t}\left({x}_{i}^{t},{y}_{i}^{t}\right)}{{D}_{i}^{t+1}\left({x}_{i}^{t},{y}_{i}^{t}\right)}\cdot\:\frac{{D}_{i}^{t}\left({x}_{i}^{t+1},{y}_{i}^{t+1}\right)}{{D}_{i}^{t+1}\left({x}_{i}^{t+1},{y}_{i}^{t+1}\right)}}_{{TC}_{i}^{t+1}}}}}\end{aligned}$$

Equation (5) is derived from Eq. (4), and its form further clarifies how TE and TC jointly contribute to variations in TFP.

It should be noted that the Malmquist index measures the growth rate of TFP between consecutive periods. Accordingly, in this study, 2006 is taken as the base year (with TFP normalized to 1), and TFP levels are constructed through cumulative multiplication across periods. This approach allows for an accurate representation of TFP across regions and years, while also facilitating panel data analysis and intertemporal comparisons. The same procedure is applied to the indicators of TE and TC.

#### Two-way fixed effects model

To examine the impact of diseases on TFP in pig farms, the following two-way fixed effects model is established:

6 $$\begin{aligned}{TFP}_{it}&=\alpha\:+{\beta{Indis}}_{it}+{\gamma{Control}}_{it}\cr&\quad+Province+Year+{\varepsilon}_{it}\end{aligned}$$

In Eq. (6), the subscript $\:i$ represents the province, and $\:t$ represents the year. $\:{TFP}_{it}$ represents TFP in pig farming; $\:{Indis}_{it}$ represents swine diseases, which is the core explanatory variable of this study; $\:{Control}_{it}$ represents the series of control variables selected in this study, including fiscal support, agricultural mechanization, livestock share, income level, and feed supply. $\:Province$ and $\:Year$ represent the province and year fixed effects, respectively; $\:\alpha\:$ is the constant term, and $\:\beta\:$ and $\:\gamma\:$ are the parameters to be estimated, while $\:{\epsilon\:}_{it}$ is the random error term.

Compared with other models, the two-way fixed effects model, by simultaneously controlling for individual fixed effects (Province) and time fixed effects (Year), can more effectively identify the impact of pig diseases on TFP. It also effectively alleviates endogeneity problems caused by omitted variables and interference from bidirectional causality. First, many variables that may simultaneously affect the risk of disease occurrence and TFP—such as inherent natural conditions of the region, long-standing farming traditions, cultural practices, and infrastructure levels—do not vary over short periods. The individual fixed effect, by introducing a unique intercept term for each province, absorbs all such time-invariant unobserved heterogeneity. The time fixed effect, by introducing a dummy variable for each year, controls for common temporal shocks. Second, the core coefficient $\:\beta\:$ estimated by the model essentially reflects the impact of within-province temporal fluctuations in disease on corresponding temporal fluctuations in TFP. This, to some extent, isolates factors that do not vary across time and individuals, thereby reducing the influence of reverse causality.

### Variable description

#### Dependent variable

The dependent variable in this study is the TFP of pig farming, which is measured using the DEA-Malmquist index. Referring to the study by Wang et al. [31], this paper selects the following input-output data to measure TFP, as shown in Table 1. The output variable is measured by the net output of the main product, calculated as the main product output minus the weight of piglets. The input variables include six main categories: piglet costs, quantity of concentrate feed, number of laborers, costs of water and fuel power, medical and preventive expenses, and depreciation of fixed assets. The variable is calculated based on the input–output data of three types of pig farms published in the “Compilation,” with TFP computed separately for each of the three farm size categories.

**Table 1:** Calculation indicators of TFP

Variable Category	Variable Name	Unit	Calculation Method
Output Variable	Net Production of Main Product	kg/head	Main Product Output- Weight of Piglets
Input Variable	Piglet Costs	Yuan/head	-
	Quantity of Concentrate Feed	kg/head	-
	Number of Laborers	Days/head	-
	Water and Fuel Costs	Yuan/head	Water Costs + Fuel Power Costs
	Medical and Preventive Expenses	Yuan/head	-
	Depreciation of Fixed Assets	Yuan/head	-

#### Independent variable

The independent variable in this study is the swine diseases, which is constructed to capture the losses caused by animal disease outbreaks. Specifically, this variable is calculated as the sum of cases, deaths, and culls reported in a given province and year for eight major swine diseases: Foot and Mouth Disease, African Swine Fever, Swine Vesicular Disease, Classical Swine Fever, Porcine Reproductive and Respiratory Syndrome, Porcine Cysticercosis, Swine Pasteurellosis, and Swine Erysipelas. The choice of this calculation method is based on two considerations. First, its economic meaning is direct. “Cases” reflect the scope of the epidemic, “Deaths” directly indicate production losses, and “Culls” represent proactive economic sacrifices made to control the spread of disease. The sum of the three comprehensively reflects the overall impact of diseases on pig farms. Second, the authority and consistency of official data. These data are derived from the Official Veterinary Bulletin regularly published by the Ministry of Agriculture and Rural Affairs of China. As the only official, publicly available, systematic, continuous, and province-disaggregated source of animal epidemic statistics at the national level, it provides an indispensable foundation for the long-term panel data analysis conducted in this study.

#### Control variables

To mitigate estimation bias caused by omitted variables, this study selects the following control variables. **(1) Fiscal Support.** Following Wang et al. [32], this is measured by the share of local government expenditure on agriculture, forestry, and water affairs in total local fiscal expenditure. A higher fiscal support level reflects stronger government commitment to agriculture, helps ease financial constraints, and promotes TFP. **(2) Agricultural Mechanization.** Following Liang et al. [33], measured by total agricultural machinery power. Mechanization enables substitution for labor and thus improves labor productivity. **(3) Livestock Share**. Referring to Yu et al. [34], measured as the share of livestock output value in the total value of agriculture, forestry, animal husbandry, and fishery services. A higher share reflects stronger livestock foundations and scale effects, enhancing TFP. **(4) Income Level**. Following Wang et al. [32], measured by the rural per capita disposable income. Rural per capita income reflects the level of economic development and purchasing power in the study area, which may affect farmers’ investment capacity, technology adoption, and overall production efficiency, thereby potentially influencing TFP. **(5) Feed Supply**. Following Wang et al. [32], measured by the share of provincial corn output in the national total. As corn is a key feed input, feed supply influences the nutrition and energy for pig growth, thus affecting TFP.

Table 2 reports the descriptive statistics of the main variables used in this study. With respect to the explanatory and control variables, because the provinces covered by different farm-size categories in the sample are largely overlapping, with only one province differing across scales, the descriptive statistics of these variables are essentially identical across farm sizes. Swine Diseases exhibits relatively large means and standard deviations, with a minimum value of 0 and a maximum of 258,139, indicating a high degree of heterogeneity in disease shocks across time and regions. Control Variables also display pronounced regional disparities, while their overall distributions remain relatively stable, allowing them to effectively capture provincial-level institutional environments and production conditions.

**Table 2:** Descriptive statistics of the variables

No.	Variables	*N*	Large-Scale				Medium-Scale				Small-Scale			
			Mean	Std. Dev.	Min	Max	Mean	Std. Dev.	Min	Max	Mean	Std. Dev.	Min	Max
1	TFP	350	1.0581	0.2256	0.5605	1.8985	1.0692	0.1932	0.7138	1.7086	1.1583	0.5154	0.4836	4.2566
2	TE	350	1.0717	0.1318	0.8210	1.4153	1.0441	0.0914	0.7850	1.2393	0.9904	0.0413	0.8297	1.0998
3	TC	350	0.9883	0.1841	0.5605	1.8300	1.0244	0.1687	0.7576	1.7651	1.1640	0.4924	0.4955	3.9396
4	Swine Diseases	350	3,922.8086	17,220.6130	0	258,139	3,901.9257	17,231.7785	0	258,139	3,901.9257	17,231.7785	0	258,139
5	Fiscal Support	350	0.1164	0.0271	0.0502	0.2038	0.1167	0.0272	0.0502	0.1897	0.1167	0.0272	0.0502	0.1897
6	Agricultural Mechanization	350	3,777.6917	2,848.93	328.53	13,353	3,719.4191	2,894.1407	328.53	13,353	3,719.4191	2,894.1407	328.53	13,353
7	Livestock Share	350	0.3050	0.0922	0.1051	0.5820	0.3079	0.0910	0.1051	0.5820	0.3079	0.0910	0.1051	0.5820
8	Income Level	350	7,264.2901	3,183.4118	2,351.96	22,099.4004	7,271.3015	3,180.5107	2,351.96	22,099.4004	7,271.3015	3,180.5107	2,351.96	22,099.4004
9	Feed Supply	350	0.0371	0.0320	0.0001	0.1451	0.0370	0.0321	0.0001	0.1451	0.0370	0.0321	0.0001	0.1451

Note: (1) For all three farm size categories, the number of sample observations is 350 (= 25 provinces × 14 years). The provinces covered by small- and medium-scale farms are identical, whereas the large-scale sample excludes Ningxia but includes Xinjiang. A list of provinces for each scale category is provided in Appendix Tables 1 and 2. (2) Due to the relatively large standard deviations of the variables Swine Diseases, Agricultural Mechanization, and Income Level, their logarithmic transformations are applied in the regression analysis. Since the Swine Diseases variable takes the value of 0 in some years, logarithmic transformation is not feasible, and these cases are treated as missing values in the regressions. Consequently, the regression sample size does not match the theoretical number of observations

## Results

### Analysis of TFP in swine production

Overall, as reported in Table 3(a), the average TFP of pig production in China during 2007–2020 was 1.095, indicating a sustained growth trend over the sample period. The corresponding mean values of TE and TC were 1.035 and 1.059, respectively, suggesting that both efficiency improvement and technological progress contributed to TFP growth, with TE playing a relatively larger role. The descriptive statistics further show that TC exhibits greater dispersion than TE, reflecting substantial heterogeneity in TC across provinces and over time, whereas TE appears comparatively more stable (Table 2).

When disaggregated by scale, small-scale pig producers exhibited the highest average TFP (1.158), followed by medium-scale and large-scale farms. This higher TFP among small-scale producers was primarily driven by strong TC, despite their relatively lower TE. In contrast, large-scale pig farms achieved the highest TE but experienced the lowest rate of TC, indicating that efficiency gains under existing technologies were their main source of productivity improvement. Medium-scale farms occupied an intermediate position, with both TE and TC jointly contributing to TFP growth. This suggests that TC was the primary driver of TFP growth for small-scale pig farms, whereas TE was the main driver for large-scale farms; for medium-scale farms, both TC and TE jointly contributed to TFP growth.

**Table 3:** TFP and cost-profit ratio of pig farms by scale

	Large-scale	Medium-scale	Small-scale	Average
(a)				
TFP	1.058	1.069	1.158	1.095
TE	1.072	1.044	0.990	1.035
TC	0.988	1.024	1.164	1.059
(b)				
Total Cost (yuan/head)	1535.277	1579.01	1609.173	1574.48
Net Profit (yuan/head)	350.50	352.92	317.85	340.42
Cost-Profit Ratio	20.60	20.64	18.29	19.84

**Fig. 3:**
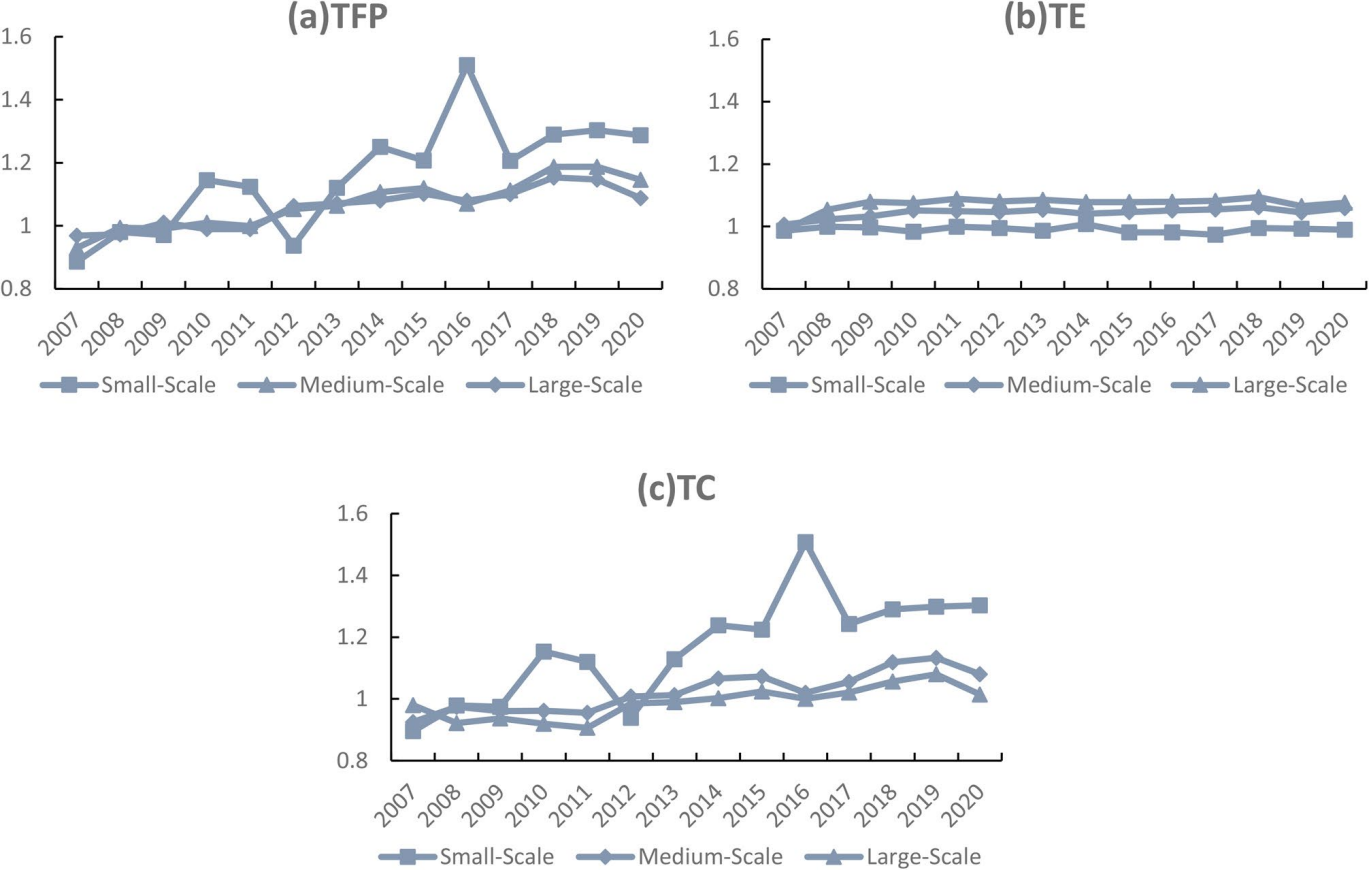
Changes in TFP of pig farms of different scales, 2007–2020

Figure 3 illustrates the changes in TFP and its components across pig farms of different scales in all provinces from 2007 to 2020. Overall, Fig. 3(a) shows that the TFP of medium- and large-scale pig farms exhibited an upward trend after 2007, followed by a decline after the outbreak of African swine fever in 2018. In contrast, the TFP of small-scale pig farms, although generally increasing, displayed greater year-to-year volatility. The reasons are as follows: on the one hand, small-scale producers employ extensive production methods, making it difficult to achieve economies of scale. Their production costs are high, and their profitability is uncertain in the face of market price fluctuations, which greatly affects their production efficiency. According to Table 3(b), the total cost of large-scale farms is 73 yuan per kilogram of main product lower than that of small-scale farms, and their cost-profit margin is about 2% points higher. On the other hand, small-scale farms have weaker risk resilience and are more vulnerable to adverse shocks such as animal disease outbreaks or changes in national policies.

Figure 3(b) illustrates the changes in TE, showing that since 2007, the TE of pig farms in China has changed little. Large-scale farms exhibited the highest TE, followed by medium-scale farms, while small-scale farms had the lowest. This may be attributed to the clear advantages of large-scale farms in capital, technology, and management, enabling them to better achieve standardized production, disease prevention, and optimized resource allocation, thereby maintaining a high and stable level of TE. By contrast, small-scale producers, constrained by insufficient capital investment, outdated management practices, and limited capacity to apply existing technologies, have persistently remained at low efficiency levels. Figure 3(c) shows the changes in TC, revealing that before 2012, the level of TC was below 1. After 2012, however, TC increased steadily, gradually becoming the main driver of TFP growth

**Fig. 4:**
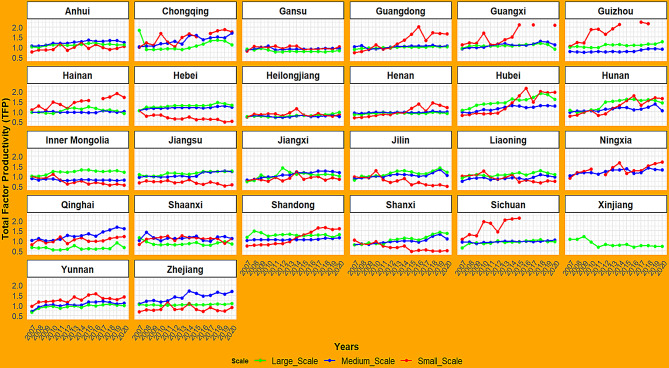
The TFP trends of pig farms by scale and province, 2007–2020

Figure 4 illustrates the changes in TFP of pig farms of different scales across all provinces from 2007 to 2020. First, in most provinces, large-scale pig farms demonstrated relatively high and stable TFP, with values generally maintained above 1.0, indicating that large-scale farming has significant advantages in TFP. Particularly in the northeastern provinces such as Jilin and Liaoning, central provinces such as Hubei and Hunan, and traditional livestock-producing regions like Shandong and Hebei, the TFP of large-scale farms was significantly higher than that of other farm sizes, reflecting the benefits of intensive production. Second, in western provinces such as Chongqing, Qinghai, and Ningxia, as well as central-eastern provinces such as Zhejiang and Anhui, medium-scale farms showed relatively high TFP, suggesting that the medium-scale farming model is more developed in these regions, likely benefiting from moderate economies of scale and sound technical management. Third, small-scale farms displayed significant regional differences in TFP, with considerable year-to-year fluctuations. In regions such as Hebei, Shaanxi, and Jiangsu, small-scale farms generally had lower TFP. However, in southwestern provinces such as Sichuan, Chongqing, and Guizhou, as well as southern provinces such as Guangdong, Guangxi, and Hainan, small-scale producers showed relatively high TFP, which may be closely related to local farming practices and policy support.

### Empirical analysis of the impact of swine diseases on TFP

#### Baseline regression

Table 4 reports the baseline regression outcomes. Controlling for year and province fixed effects, swine diseases exhibit markedly heterogeneous effects on farm productivity across scales. Epidemics significantly enhance the TFP of large-scale farms but significantly reduce that of small-scale farms, whereas the impact on medium-scale farms is statistically insignificant. Quantitatively, a 1% rise in epidemic incidence corresponds to a 0.018-unit increase in TFP for large-scale farms and a 0.041-unit decrease for small-scale farms.

**Table 4:** Baseline regression results

	Large-Scale		Medium-Scale		Small-Scale	
	(1)	(2)	(3)	(4)	(5)	(6)
Swine Diseases	0.0180^**^	0.0180^***^	0.0016	0.0013	-0.048^***^	-0.041^***^
	(0.0074)	(0.0063)	(0.0044)	(0.0042)	(0.0131)	(0.0121)
Control Variables	NO	YES	NO	YES	NO	YES
Time Fixed Effects	YES	YES	YES	YES	YES	YES
Province Fixed Effects	YES	YES	YES	YES	YES	YES
Constant	-0.1962^***^	0.0830	-0.0837^*^	-1.6631	0.2540^*^	-12.8390^**^
	(0.0621)	(3.0933)	(0.0460)	(2.8093)	(0.1296)	(5.5216)
*N*	322	322	322	322	322	322

Note: This table reports regression results for three farm sizes (large-, medium-, and small-scale). Columns (1), (3), and (5) present results without control variables, while Columns (2), (4), and (6) include control variables. Cluster-robust standard errors are reported in parentheses. *, **, and *** denote significance at the 10%, 5%, and 1% levels, respectively. Same notes apply to subsequent tables

#### Robustness checks

To test the robustness of the baseline regression results, this study replaces the original explanatory and explained variables with the lagged epidemic incidence level and the TFP of pig farming estimated by the SFA method, respectively. From the perspective of lagged epidemic incidence, swine diseases not only affect producers’ decisions in the current period but also exert an impact on their subsequent production behavior, for example by adjusting production plans or increasing investments in biosecurity, thereby influencing TFP. The regression results show in Table 5, consistent with the baseline findings, the lagged epidemic incidence level exerts a positive effect on the TFP of large-scale farms, a negative effect on small-scale farms, and no significant effect on medium-scale farms.

Unlike the DEA method, the SFA approach requires the specification of a production function, which allows for the separation of random errors and technical inefficiency. Following Wang et al. [31], this study adopts the Translog production function for pig farming and estimates TFP based on this framework. The TFP results obtained from DEA and SFA methods validate each other, further confirming the robustness and reliability of the findings. As shown in Table 5, the regression outcomes based on SFA-TFP are consistent with the baseline regression results in both significance level and direction of impact, thereby providing strong evidence of robustness.

**Table 5:** Robustness checks results

	Large-Scale		Medium-Scale		Small-Scale	
	DEA-TFP	SFA-TFP	DEA-TFP	SFA-TFP	DEA-TFP	SFA-TFP
L.Dis	0.0117^**^		0.0010		-0.0208^*^	
	(0.0055)		(0.0034)		(0.0101)	
Swine Diseases		0.0059^*^		-0.0021		-0.0067^**^
		(0.0029)		(0.0018)		(0.0032)
Control Variables	YES	YES	YES	YES	YES	YES
Time Fixed Effects	YES	YES	YES	YES	YES	YES
Province Fixed Effects	YES	YES	YES	YES	YES	YES
Constant	-1.4875	4.666^***^	-0.6856	-5.1755^***^	-11.8384^*^	4.6382^***^
	(2.8008)	(1.1082)	(3.0936)	(2.8093)	(6.3002)	(0.0199)
*N*	297	320	297	322	297	322

#### Mechanism analysis

Table 6 reports the regression results using TE and TC as mechanism variables. It can be observed that, except for medium-scale farms, swine diseases have no significant impact on TE but exert a significant influence on TC. Specifically, a 1% increase in epidemic incidence raises the TC of large-scale farms by 0.0141 units, while reducing that of small-scale farms by 0.0415 units.

For large-scale farms, epidemic shocks stimulate enterprises to increase R&D investment, adopt more advanced vaccines, and implement intelligent farming technologies, thereby driving TC across all production stages and ultimately enhancing TFP. In contrast, small-scale farms experience destructive effects from epidemic shocks. Due to limited financial resources, lack of technical support, and weak resilience, small-scale farmers are forced to exit the market and fail to achieve technological upgrading. For medium-scale farms, swine diseases significantly improve TE. This suggests that medium-scale farms respond to epidemic shocks by optimizing production management practices—such as strengthening existing disease prevention measures and improving farming environments—thus achieving efficiency gains.

**Table 6:** Mechanism analysis results

	Large-Scale		Medium-Scale		Small-Scale	
	TE	TC	TE	TC	TE	TC
Swine Diseases	0.0039	0.0141^**^	0.0046^***^	-0.0032	0.0005	-0.0415^***^
	(0.0025)	(0.0056)	(0.0014)	(0.0036)	(0.0013)	(0.0121)
Control Variables	YES	YES	YES	YES	YES	YES
Time Fixed Effects	YES	YES	YES	YES	YES	YES
Province Fixed Effects	YES	YES	YES	YES	YES	YES
Constant	0.5196	-0.4124	-0.9792	-0.6597	-0.0719	-12.7358^**^
	(1.1141)	(2.4563)	(0.8714)	(2.5334)	(0.4742)	(5.4933)
N	322	322	322	322	322	322

#### Heterogeneity analysis

**Fig. 5:**
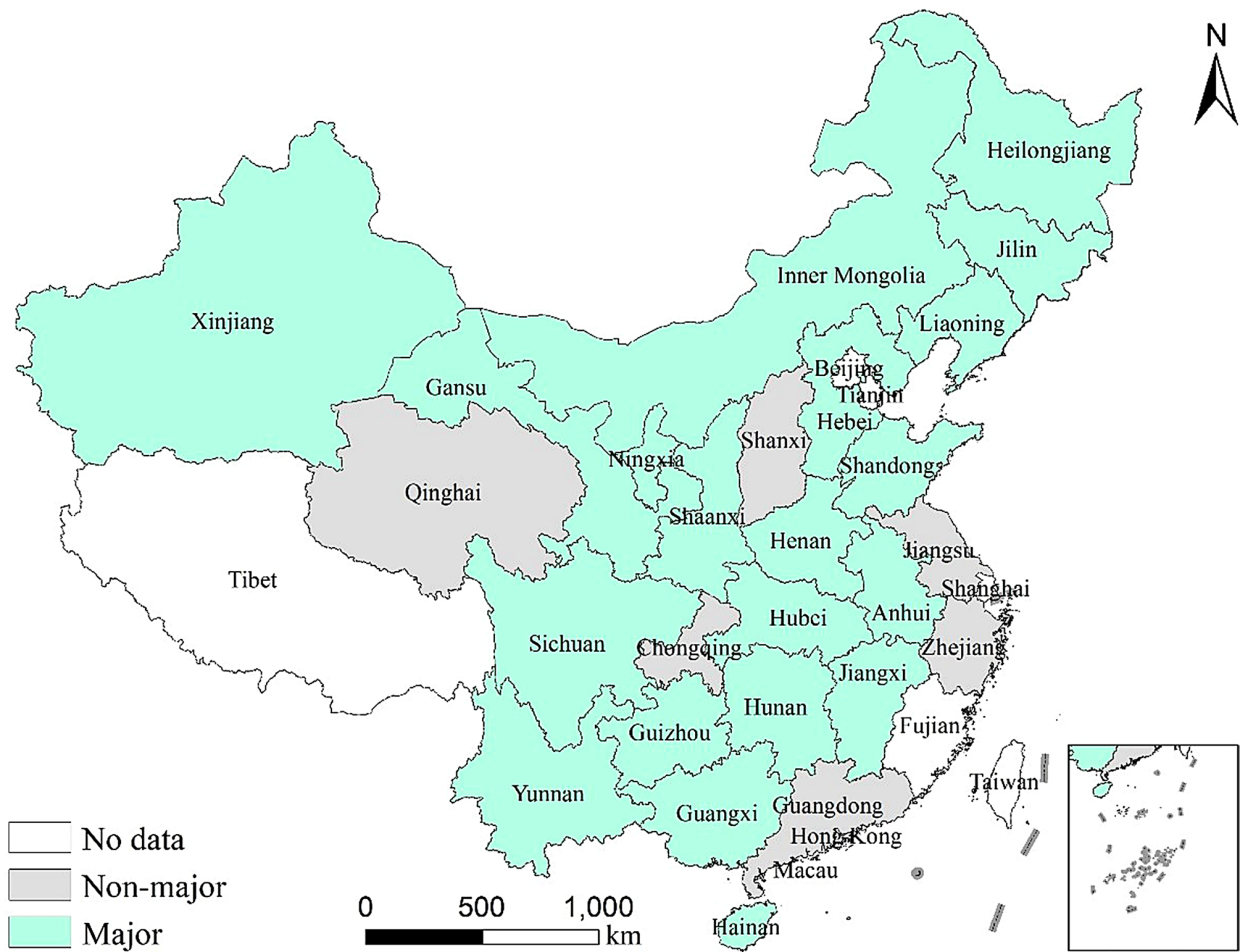
Distribution of sample provinces classified as major and non-major production areas. Map Review Number: GS(2024)0650

Overall, the impact of swine diseases on TFP exhibits substantial heterogeneity across different production regions. Taking major production areas and non-major production areas as examples, significant differences in resource endowments, industrial structures, policy support, and market environments may shape the effect of diseases on TFP in pig farming. Following Pan et al. [35], this study identifies major and non-major production areas based on the relationship between provincial per capita pork consumption and per capita pork production in 2020 (See Fig. 5). If a province’s per capita pork consumption is lower than its per capita production, the province has a surplus of pork and must supply other regions, thus it is classified as a major production area; otherwise, it is classified as a non-major production area.

The results show that the impact of swine diseases on TFP differs markedly between major and non-major production areas (Table 7). For large-scale farms, disease shocks have a significantly positive effect on TFP in major production areas, while the effect is not significant in non-major areas. Medium-scale farms experience a significant positive impact in non-major areas, whereas the coefficient is negative but not significant in major areas, indicating that medium-scale farms in non-major regions benefit from lighter disease pressure but face higher production risks in major regions. In contrast, small-scale farms suffer significant negative effects in both types of regions, with a larger impact in non-major areas, suggesting that in places lacking coordinated disease control and resource support, small producers are less able to withstand the efficiency losses caused by diseases.

**Table 7:** Heterogeneity analysis results

	Large-Scale		Medium-Scale		Small-Scale	
	Non-Major Area	Major Area	Non-Major Area	Major Area	Non-Major Area	Major Area
Swine Diseases	0.0128	0.0170^*^	0.0168^*^	-0.0010	-0.0707^**^	-0.0387^**^
	(0.0109)	(0.0091)	(0.0080)	(0.0040)	(0.0245)	(0.0152)
Control Variables	YES	YES	YES	YES	YES	YES
Time Fixed Effects	YES	YES	YES	YES	YES	YES
Province Fixed Effects	YES	YES	YES	YES	YES	YES
Constant	3.1495	-0.3419	-7.8107^***^	-0.2370	-17.8225^**^	-7.6274
	(2.7383)	(4.0729)	(1.5974)	(2.8009)	(6.0074)	(7.5751)
N	76	246	76	246	76	246

## Discussion

This study finds that between 2007 and 2020, the TFP of pig farms in China continued to increase across different farm sizes. During this period, the TE did not show a marked improvement, whereas TC exhibited a growth trend consistent with that of TFP. The average TE of Chinese pig farms was 1.035, which is higher than that reported in other countries. For instance, the average TE of pig farms in Catalonia, Spain, was 0.94 [36], while that of pork producers in Denmark and Poland was 0.899 and 0.859, respectively [37]. In addition, the average level of TC in Chinese pig farms was 1.059, contributing more to TFP growth than TE. From the perspective of farm size, small-scale farms exhibited lower TE, a finding consistent with Singbo et al.’s study on pig production in Quebec [21].

The results of this study indicate that the impact of swine diseases on the TFP of pig farms in China exhibits significant scale heterogeneity. TFP in large-scale farms increases significantly following disease shocks, whereas that of small-scale producers decreases markedly, while medium-scale producers show no statistically significant effect. This finding is broadly consistent with existing research. First, large agricultural enterprises typically possess stronger capacities for resource integration and risk management, which enable them to transform external negative shocks into opportunities for efficiency gains. For example, the outbreak of African swine fever generated average abnormal returns of 10% to 40% for listed pig farming companies in China, with larger enterprises reaping more substantial market benefits [38]. Such positive feedback from capital markets provides firms with resources and incentives for further development, encouraging increased technological investment and improved management models, which ultimately promote TFP growth. In contrast, small-scale farmers, constrained by limited capital and technology, are more prone to production decline when confronted with disease outbreaks [39]. Second, the resilience demonstrated by large-scale enterprises in the face of swine diseases further supports the hypothesis of “induced TC”. For large-scale farms, disease outbreaks render pig assets scarce and costly. In response, these farms, leveraging their capital reserves, technological accumulation, and economies of scale, develop novel biosecurity technologies and adapt farm management practices, thereby driving improvements in TFP.

Moreover, existing empirical studies on this issue remain limited, particularly with respect to scale heterogeneity, which constrains the comparability and scope of discussion in this study. Previous evidence shows that a 1% increase in the incidence of zoonotic diseases reduces TFP in the livestock sector by 0.022–0.036% points [29], a trend broadly consistent with the findings of this study. Other research further suggests that the outbreak of African swine fever in China significantly reduced overall production efficiency [40], which partially aligns with this study’s results in that animal diseases lead to efficiency losses among small-scale pig farms. The main reason for this discrepancy lies in the fact that earlier studies did not systematically examine heterogeneity across different farm scales, thereby overlooking the resilience and efficiency-enhancing potential exhibited by large-scale farms when confronted with epidemic shocks.

From the perspective of impact mechanisms, TC is the key factor through which animal diseases affect TFP in the pig industry. This finding is consistent with the situation observed in other countries. For instance, in France, the early growth of pig farm TFP was driven by improvements in TE, followed later by TC [41]. In Spain and Poland, the continuous growth of pig farm TFP has also been mainly attributed to TC [19, 37]. On the other hand, the TE, as a reflection of resource allocation efficiency and management capability, also plays an important role in improving TFP. This study finds that diseases significantly improved the TE of medium-scale pig farms, but had no significant effect on farms of other scales. This result contrasts with Yan et al., who concluded that ASF reduced the TE of large-scale pig farms [28]. Two main reasons account for this divergence: first, this study employs an econometric model that rigorously controls for confounding factors, whereas Yan et al. relied on a simple comparison of TE before and after the outbreak of ASF. Second, this study covers ten major pig diseases, including African swine fever, and uses a 12-year panel dataset, which enables a more comprehensive assessment of the long-term impacts of animal diseases on TE.

The findings of this study provide the following implications for the development of the pig industry in China and other countries. First, the heterogeneity of pig farms across different scales should be fully recognized. For large-scale farms, the priority should be to optimize managerial capacity and resource allocation efficiency, so as to further harness the potential of TC [42]. For small- and medium-scale farms, efforts should focus on enhancing their risk resilience and ability to adopt new technologies, in order to prevent them from falling into sustained production decline under epidemic shocks. Second, the technological level of pig farming should be improved. On the one hand, investments in breeding research and development should be increased, and modern biotechnologies such as molecular breeding and genomic selection should be actively applied to cultivate superior breeds, thereby improving herd health and production performance. On the other hand, precision livestock farming should be actively promoted, including the adoption of intelligent precision feeding systems, which can improve animal welfare and feed efficiency while reducing human–animal contact frequency, thus lowering disease transmission risks and enhancing economic benefits [43]. Third, the TE of pig farms should be strengthened. This can be achieved through promoting efficient management models and improving standardized production systems, thereby continuously raising resource allocation and farm management capabilities and ultimately achieving sustained improvements in TE.

Despite providing a comprehensive analysis of the impact of animal diseases on TFP in pig farming, this study has certain limitations. First, the heterogeneity across different diseases is not explicitly addressed. Distinct pig diseases vary substantially in terms of transmission mechanisms, mortality rates, and the intensity of government interventions, which may lead to heterogeneous impacts on TFP. This limitation primarily stems from the insufficient availability of disaggregated epidemiological data. Existing data are largely confined to the provincial level, and differences in local governments’ disclosure of sensitive disease information create additional barriers to disease-specific assessments. This challenge is also common in animal health economics research. Accordingly, we advocate that governments should strengthen the collection and disclosure of animal disease data to provide essential support for both academic research and livestock industry development. Second, the dataset employed in this study consists of aggregated provincial-level statistics, which, while authoritative and representative, cannot fully capture farm-level decision-making processes and management practices. For example, changes in TE may result from specific biosecurity measures or husbandry adjustments, but such details are difficult to observe in macro-level data. Future research could therefore benefit from incorporating survey-based micro data or firm-level panel data to complement and validate the findings of this study. Moreover, we acknowledge that international trade may influence swine disease outbreaks and TFP. However, due to its limited direct impact on pig farms in China, trade variables were not included in this study. Future research could consider collecting more trade-related data to investigate the relationship between trade, TFP, and disease spread.

## Conclusion

This study yields the following main conclusions. First, swine diseases exert heterogeneous impacts on the TFP of pig farms across different scales. After controlling for province and year fixed effects as well as other relevant covariates, the results show that disease outbreaks significantly increase the TFP of large-scale pig farms, significantly decrease the TFP of small-scale farms, and exert no significant effect on medium-scale farms. Specifically, a 1% increase in the number of disease raises the TFP of large-scale pig farms by an average of 0.018 units, while reducing the TFP of small-scale farms by an average of 0.041 units. Second, the mechanisms through which diseases affect TFP vary by farm size. For large- and small-scale farms, the impact primarily operates through changes in TC, while for medium-scale farms, diseases improve TE without significantly influencing TC. Third, there is pronounced regional heterogeneity in the effects of diseases. In major production areas, where pig farming is more intensive and industrially agglomerated, the impact of diseases on TFP across different farm scales is generally stronger than in non-major production areas. Overall, by uncovering the differentiated effects and mechanisms of animal diseases on pig farms of varying scales, this study provides new insights into the sources of efficiency differences in the pig industry, and offers empirical evidence for formulating scale-specific policies aimed at promoting the sustainable development of the sector.

## Supplementary Information

Below is the link to the electronic supplementary material.

Supplementary Material 1

## Data Availability

Data on animal diseases were obtained from the Official Veterinary Bulletin (available at: https://www.moa.gov.cn/gk/sygb/). The Compilation of National Agricultural Product Cost-Benefit Data is only available in printed form and can be obtained from the corresponding author upon request. Other data were sourced from various official statistical yearbooks and are also available from the corresponding author upon request.

## Footnotes

1 Based on the authors’ own calculations using publicly available data from the National Bureau of Statistics of China.

2 Based on the authors’ own calculations using publicly available data from the Ministry of Agriculture and Rural Affairs of China.

3 Due to significant differences in pig farm scales across provinces, not all provinces have production data for all farm sizes in every year. Therefore, in the regression analysis, the samples for different farm sizes cover only 25 provinces.

## Abbreviations

ASF: African Swine Fever
PED: Porcine Epidemic Diarrhea
PRRS: Porcine Reproductive and Respiratory Syndrome
TFP: Total Factor Productivity
TE: Technical Efficiency
TC: Technological Change
SFA: Stochastic Frontier Approach
