# Supplementary material for: The impact of swine diseases on total factor productivity of pig farms of different scales in China
Source: Porcine Health Manag. 2026 Jan 24;12:9. doi: 10.1186/s40813-025-00484-z (PMC12937567; doi:10.1186/s40813-025-00484-z)
Supplement: Supplementary file 1 — Supplementary Material 1 [file 40813_2025_484_MOESM1_ESM.docx]

1. Appendix Table 1 presents the provincial mean values of the main variables used in the analysis. “Swine Diseases” records the average annual number of reported cases, deaths, and culls from major swine diseases. “Fiscal Support” is the share of local government expenditure on agriculture, forestry, and water affairs in total fiscal spending. “Agricultural Mechanization” refers to total agricultural machinery power. “Livestock Share” measures the share of livestock output in total agricultural output. “Income Level” is the per capita disposable income of rural residents. “Feed Supply” represents each province’s corn production as a share of the national total. These summary statistics provide an overview of the production and economic environment across the sample provinces.

**Appendix Table 1.** Mean values of major variables by province

| Province | Swine Diseases | Fiscal Support | Income Level | Agricultural Mechanization | Livestock Share | Feed Supply |
| --- | --- | --- | --- | --- | --- | --- |
| Hebei | 5.3838 | 0.1109 | 7235.7514 | 9347.7814 | 0.3495 | 0.0367 |
| Shanxi | 3.4304 | 0.1138 | 6323.2257 | 2420.9943 | 0.2704 | 0.0128 |
| Inner Mongolia | 4.3024 | 0.1424 | 7352.0386 | 3331.1943 | 0.4352 | 0.0071 |
| Liaoning | 4.2104 | 0.0875 | 7955.2386 | 2352.2329 | 0.3708 | 0.0430 |
| Jilin | 3.6472 | 0.1260 | 7397.1800 | 2767.3164 | 0.4675 | 0.0195 |
| Heilongjiang | 4.7333 | 0.1455 | 7045.1500 | 4836.1379 | 0.3247 | 0.0285 |
| Jiangsu | 5.8908 | 0.0944 | 11035.0243 | 4443.8929 | 0.1970 | 0.0312 |
| Zhejiang | 6.7265 | 0.0918 | 14931.3243 | 2258.5557 | 0.1792 | 0.0285 |
| Anhui | 6.2312 | 0.1069 | 7314.4021 | 5977.3200 | 0.3034 | 0.0179 |
| Jiangxi | 7.8643 | 0.1191 | 7458.2386 | 2840.3307 | 0.2791 | 0.0604 |
| Shandong | 4.5871 | 0.1037 | 8663.0664 | 11335.0857 | 0.2788 | 0.0757 |
| Henan | 5.6514 | 0.1086 | 7069.8600 | 10343.2557 | 0.3173 | 0.0798 |
| Hubei | 8.7687 | 0.1082 | 7646.6193 | 3865.7871 | 0.2651 | 0.0388 |
| Hunan | 8.2448 | 0.1135 | 7347.1857 | 5399.0136 | 0.3261 | 0.0925 |
| Guangdong | 7.5707 | 0.0601 | 9451.4843 | 2390.3271 | 0.2428 | 0.1222 |
| Guangxi | 8.9347 | 0.1225 | 6290.2007 | 3248.5657 | 0.2894 | 0.0602 |
| Hainan | 5.3862 | 0.1370 | 6697.2921 | 487.6343 | 0.1935 | 0.0158 |
| Chongqing | 8.8621 | 0.0844 | 6982.1864 | 1207.7321 | 0.3141 | 0.0177 |
| Sichuan | 8.3675 | 0.1134 | 6896.6443 | 3834.5771 | 0.4040 | 0.0715 |
| Guizhou | 6.6589 | 0.1379 | 4876.2200 | 2084.6050 | 0.2811 | 0.0107 |
| Yunnan | 7.7173 | 0.1373 | 5534.4621 | 2766.9536 | 0.3413 | 0.0245 |
| Shaanxi | 6.2596 | 0.1150 | 5549.1529 | 2197.8379 | 0.2535 | 0.0212 |
| Gansu | 5.9523 | 0.1499 | 4352.7264 | 2115.5214 | 0.1785 | 0.0065 |
| Qinghai | 4.8012 | 0.1321 | 4623.7636 | 432.4221 | 0.5304 | 0.0005 |
| Ningxia | 4.7181 | 0.1548 | 5754.1000 | 700.4036 | 0.3042 | 0.0017 |
| Xinjiang | 6.2310 | 0.1480 | 5578.8157 | 2157.2164 | 0.2328 | 0.0041 |

2. Appendix Table 2 presents the average values of TFP, TE, and TC for pig farms of different scales across the sampled provinces. The statistics summarize the productivity level, efficiency performance, and technological progress of farms, providing insights into the heterogeneity of production efficiency among regions and scales.

**Appendix Table 2.** Average TFP, TE, and TC of Pig Farms of Different Scales in Each Province

|  | Large-scale | | | Medium-Scale | | | Small-Scale | | |
| --- | --- | --- | --- | --- | --- | --- | --- | --- | --- |
| Province | TFP | TE | TC | TFP | TE | TC | TFP | TE | TC |
| Hebei | 1.2900 | 1.3951 | 0.9256 | 1.1967 | 1.2282 | 0.9747 | 0.6966 | 0.9935 | 0.7014 |
| Shanxi | 1.0691 | 0.9846 | 1.0830 | 0.9931 | 1.1486 | 0.8632 | 0.6896 | 1.0049 | 0.6851 |
| Inner Mongolia | 1.2065 | 1.3417 | 0.9009 | 0.8269 | 0.9938 | 0.8314 | 0.7534 | 0.9930 | 0.7585 |
| Liaoning | 1.0947 | 1.0630 | 1.0316 | 0.9146 | 0.9862 | 0.9277 | 0.9073 | 0.9829 | 0.9207 |
| Jilin | 1.1341 | 0.9970 | 1.1367 | 1.0498 | 1.0348 | 1.0123 | 0.7740 | 1.0029 | 0.7705 |
| Heilongjiang | 0.8101 | 0.9484 | 0.8544 | 0.7759 | 0.9570 | 0.8110 | 0.8699 | 0.9681 | 0.8994 |
| Jiangsu | 1.1478 | 1.1325 | 1.0146 | 1.0748 | 0.9830 | 1.0891 | 0.6876 | 0.9718 | 0.7063 |
| Zhejiang | 1.0421 | 1.0896 | 0.9576 | 1.4237 | 0.9874 | 1.4469 | 0.8383 | 0.9657 | 0.8673 |
| Anhui | 1.1126 | 1.1729 | 0.9495 | 1.2234 | 1.1822 | 1.0335 | 0.9704 | 0.9838 | 0.9852 |
| Jiangxi | 1.0549 | 0.9702 | 1.0846 | 1.0805 | 0.9925 | 1.0865 | 0.8884 | 0.9798 | 0.9056 |
| Shandong | 1.2975 | 1.1492 | 1.1288 | 1.0729 | 1.0759 | 0.9988 | 1.1612 | 0.9761 | 1.1860 |
| Henan | 0.9235 | 1.2342 | 0.7484 | 0.9673 | 1.1663 | 0.8286 | 1.0133 | 0.9649 | 1.0502 |
| Hubei | 1.5066 | 1.2524 | 1.2001 | 1.1720 | 1.1081 | 1.0580 | 1.3797 | 0.9780 | 1.4073 |
| Hunan | 1.3696 | 1.0000 | 1.3696 | 1.1231 | 1.0000 | 1.1231 | 1.2436 | 0.9756 | 1.2763 |
| Guangdong | 0.9828 | 1.0465 | 0.9386 | 1.0233 | 0.9840 | 1.0397 | 1.2795 | 0.9784 | 1.3089 |
| Guangxi | 1.0711 | 0.9937 | 1.0773 | 1.0974 | 1.0224 | 1.0722 | 1.7804 | 1.0634 | 1.6753 |
| Hainan | 1.0603 | 0.9724 | 1.0918 | 0.9898 | 0.9668 | 1.0256 | 1.5451 | 1.0270 | 1.5064 |
| Chongqing | 1.1085 | 0.9973 | 1.1092 | 1.3330 | 1.2161 | 1.0920 | 1.5248 | 0.9737 | 1.5641 |
| Sichuan | 0.9309 | 0.9880 | 0.9392 | 0.9634 | 0.9940 | 0.9696 | 2.1428 | 1.0539 | 2.0320 |
| Guizhou | 1.0881 | 1.1593 | 0.9367 | 0.8092 | 0.9849 | 0.8212 | 2.0005 | 1.0031 | 1.9982 |
| Yunnan | 0.9476 | 0.9877 | 0.9582 | 1.0453 | 1.0001 | 1.0423 | 1.2987 | 0.9951 | 1.3009 |
| Shaanxi | 0.8817 | 0.9868 | 0.8922 | 1.1405 | 1.0000 | 1.1405 | 1.1006 | 0.9626 | 1.1428 |
| Gansu | 0.8167 | 0.9591 | 0.8513 | 0.9224 | 0.9461 | 0.9739 | 0.9637 | 0.9782 | 0.9839 |
| Qinghai | 0.6511 | 0.9916 | 0.6565 | 1.2749 | 1.1454 | 1.1120 | 1.0347 | 0.9891 | 1.0447 |
| Ningxia |  |  |  | 1.2357 | 0.9994 | 1.2365 | 1.4136 | 0.9941 | 1.4223 |
| Xinjiang | 0.8537 | 0.9782 | 0.8708 |  |  |  |  |  |  |
